# An Atypical Presentation of ST-Segment Elevation Myocardial Infarction in a 30-Year-Old Male Patient: A Case Report

**DOI:** 10.7759/cureus.93586

**Published:** 2025-09-30

**Authors:** Waleed Bandey, Rujina Begum, Marwa Khan, Taylor Davis

**Affiliations:** 1 Emergency Medicine, Midland Metropolitan University Hospital, Birmingham, GBR; 2 Acute Medicine, Midland Metropolitan University Hospital, Birmingham, GBR

**Keywords:** acs, acute coronary syndrome, cardiovascular disease, ecg, mi, myocardial infarction, stemi, st-segment elevation myocardial infarction (stemi), troponin

## Abstract

Myocardial infarction (MI) occurs when there is a significant reduction or obstruction in coronary blood flow, resulting in necrosis of the myocardial tissue. This case report describes a rare presentation of ST-segment elevation MI (STEMI) in a young patient without significant medical or familial risk factors. It highlights four key aspects: the presentation of MI in a young patient, the atypical nature of the chest pain, the importance of measuring troponin levels even when the electrocardiogram (ECG) appears normal, and the critical role of serial ECGs in diagnosing MI.

## Introduction

Myocardial infarction (MI) occurs when there is a significant reduction or cessation of blood flow to a coronary artery, leading to necrosis of myocardial tissue. The most common symptom is retrosternal chest pain or discomfort, which typically radiates to the left shoulder, arm, or jaw and is often described as pressure, tightness, or heaviness. Additional symptoms can include shortness of breath, nausea, light-headedness, and diaphoresis [1]. Patients can also experience silent MIs. For example, a study has shown that in diabetic patients, the prevalence of silent MI is around 4%, rising to approximately 30% in those with established coronary or peripheral arterial disease [2].

The majority of MIs are attributed to coronary artery disease. Risk factors include hypertension, diabetes mellitus, hyperlipidaemia, tobacco use, sedentary lifestyle, obesity, poor dietary habits, and excessive alcohol consumption [3,4]. The most frequent cause of coronary heart disease is the rupture of an atherosclerotic plaque, leading to blockage of a coronary artery [3]. Less commonly, MIs may result from coronary artery spasms, which can be triggered by substances such as cocaine, significant emotional stress (often referred to as takotsubo syndrome), or extreme cold exposure [3,5]. Diagnostic tools include electrocardiogram (ECG), cardiac biomarkers such as troponin, and clinical history. 

Timely treatment of MI is critical. Aspirin is an appropriate immediate intervention in suspected cases [6]. Nitrates or opioids may be administered to alleviate chest pain; however, these do not improve overall outcomes [7]. Opioids are reserved for patients experiencing severe pain. Nitrates are particularly beneficial in cases of ongoing chest pain or hypertension; however, they should be used cautiously in hypotensive patients. Supplemental oxygen is recommended for those with hypoxia. Once ST-segment elevation MI (STEMI) is confirmed, eligibility for percutaneous coronary intervention (PCI) must be assessed. PCI should be performed when patients present within 12 hours of symptom onset and within 120 minutes of the time fibrinolysis could have been initiated. However, if patients present after 12 hours and still show signs of ongoing ischaemia, PCI should still be considered. Additional antiplatelet therapy is also advised before PCI. Fibrinolysis should be initiated if PCI is unavailable or unfeasible within the recommended timeframe [6]. Following an MI, lifestyle modifications and long-term management with antiplatelet agents, angiotensin-converting enzyme inhibitors, beta-blockers, and statins are generally recommended. 

We present a case of a 30-year-old male who developed an acute STEMI. Our case highlights the need for awareness of atypical presentations with respect to patient age and symptomatology, to avoid delayed diagnosis and treatment.

## Case presentation

A 30-year-old patient presented to the accident & emergency (A&E) department with chest pain that had begun approximately four to five hours earlier, while at home. This was his first presentation for chest pain. The pain was described as sharp and pleuritic, radiating to both arms with a pins-and-needles sensation, and accompanied by mild shortness of breath. The episodes were intermittent, each lasting about 10 minutes. The patient reported no recent infections, no significant past medical history, and no family history of cardiovascular disease. He was a non-smoker and denied substance use. 

On examination, the patient appeared clammy. No murmurs or other abnormal clinical findings were identified. 

**Figure 1:**
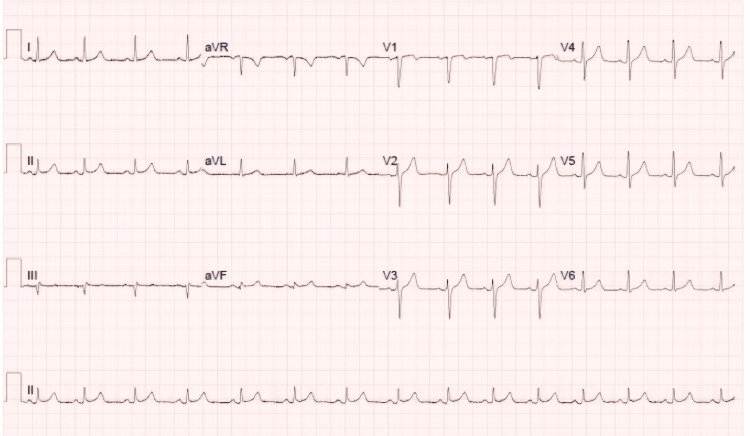
The first ECG done upon presentation to the accident & emergency department.

**Figure 2:**
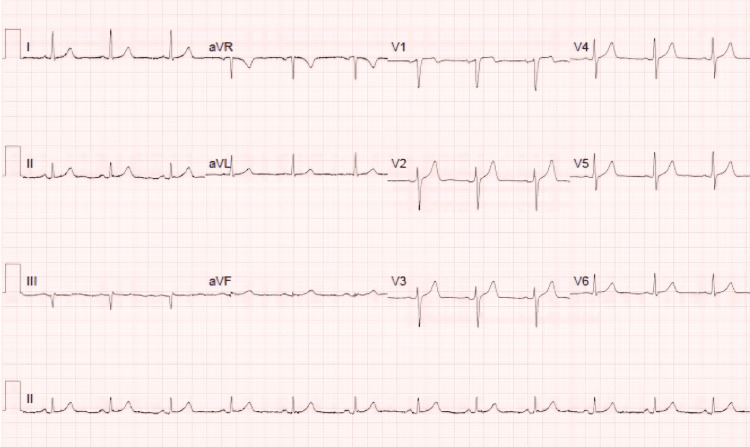
A second ECG, repeated after 90 minutes, showing no ischaemic changes. A second ECG, repeated after 90 minutes, showing no ischaemic changes.

Serial ECGs and routine blood tests, including troponin levels, were performed. The initial ECG (Figure 1) was unremarkable; however, the troponin levels were markedly elevated at 890 ng/L. A repeat ECG (Figure 2), conducted approximately 90 minutes later due to recurrent chest pain, also showed no abnormalities. The patient's vital signs remained stable, and he was transferred to a monitored bed due to the elevated troponin. A second troponin sample was taken while awaiting cardiology review, and the acute coronary syndrome (ACS) protocol was initiated. 

**Figure 3:**
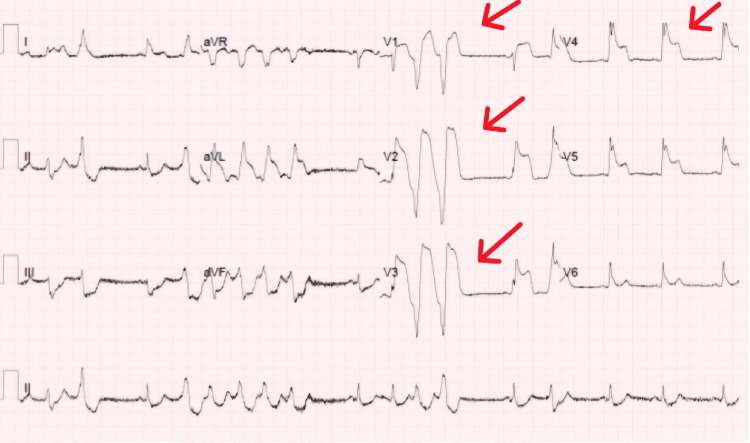
The third ECG, done 90 minutes after the second, showed ST-segment elevation with reciprocal changes and non-sustained ventricular tachycardia.

Approximately 90 minutes later, the patient experienced another episode of chest pain, prompting a third ECG (Figure 3), which revealed ST elevation and non-sustained ventricular tachycardia. He was promptly transferred to the cardiac catheterisation laboratory, where coronary angiography revealed a subtotal thrombotic occlusion of the proximal left anterior descending artery, accompanied by mild mid- and distal atheroma. PCI was subsequently performed on the left anterior descending artery. A post-angiogram echocardiogram revealed an ejection fraction of 45%. Table 1 shows the patient’s troponin and lipid levels. The remaining blood tests were unremarkable.

**Table 1:** The patient's laboratory findings The results of blood investigations performed upon presentation to the accident & emergency department showed significantly raised troponin levels. HDL: high-density lipoprotein; LDL: low-density lipoprotein

Blood test	Value	Normal range
First troponin	890 ng/L	0-4.9 ng/L
Second troponin	1272.8 ng/L	0-4.9 ng/L
Lipoprotein (a)	6.1 nmol/L	<75.0 nmol/L
Total cholesterol	5.6 mmol/L	<5.0 mmol/L
HDL cholesterol	1.0 mmol/L	>1.0 mmol/L (men), >1.2 mmol/L (women)
Cholesterol to HDL ratio	5.6 mmol/L	<6.0 mmol/L
LDL cholesterol	3.9 mmol/L	<3.0 mmol/L
Non-HDL cholesterol	4.6 mmol/L	<4.0 mmol/L
Triglyceride level	1.6 mmol/L	<1.7 mmol/L

## Discussion

Chest pain in a relatively younger population may not always follow the classical predictive presentation of ACS. This variability can lead to delays in instituting gold-standard treatment such as PCI. In this case, symptoms were atypical, and the initial ECG findings were not strongly indicative of an acute MI. However, elevated troponin levels suggested an ischaemic cardiac event, which was later confirmed as the patient developed characteristic ECG changes consistent with MI, necessitating urgent coronary intervention.

The Office for National Statistics (UK) documented 20,557 deaths from acute MI in England and Wales in 2022, of which 19 occurred in individuals aged 0-29 years [8]. In addition, data from the Framingham Heart Study’s 10-year follow-up indicated that the incidence of MI per 1000 individuals in men aged 30 to 34, 35 to 44, and 45 to 54 years was 12.9, 38.2, and 71.2, respectively. For women, the figures were 2.2, 5.2, and 13.0 in the same age groups [9]. Although less common, the possibility of MI in a young patient should not be overlooked, and these figures highlight the need for awareness.

Early diagnosis of MI is crucial for any patient, and this case highlights several key points. It emphasises the necessity of measuring troponin levels even when the ECG appears normal, particularly if the symptoms have cardiac characteristics. Elevated troponin levels in a young patient can also suggest conditions such as pericarditis or myocarditis. Myopericarditis in young patients presenting with retrosternal pain, elevated troponin, and ECG findings showing widespread ST elevation has been seen [10]. Acute pericarditis is associated with saddle-shaped ST elevation, with PR depression being the most specific marker of the disease. Patients may exhibit other symptoms, for example, chest pain, which may be pleuritic and relieved by sitting forward. They can have associated flu-like symptoms, dyspnoea and a non-productive cough. Patients may also have a pericardial rub. Patients with myocarditis may also present with chest pain and shortness of breath. This can be associated with arrhythmias and ST and T wave changes, including ST-segment elevation and T wave inversion. 

It is important to acknowledge the potential for atypical chest pain presentations in patients experiencing MI. In a retrospective cohort study, Coventry et al. reported that 26% of 382 patients diagnosed with MI presented without chest pain [11]. In our case, the patient exhibited sharp, pleuritic pain rather than the typical heavy pressure sensation commonly associated with MI. This highlights the variability in MI presentation and underscores the importance of considering atypical symptoms in diagnosis. Studies have demonstrated that increasing age, female gender, and comorbidities such as diabetes mellitus are associated with an increased risk of atypical or absent chest pain [12]. It is therefore crucial to consider these factors when assessing patients. 

Moreover, our case highlights the importance of conducting serial ECGs, as the characteristic ECG pattern indicative of STEMI emerged approximately nine hours after symptom onset, on the third ECG. As recommended by Ayer et al., serial ECGs should be performed when the initial ECG is non-diagnostic but the patient’s signs or symptoms remain consistent with acute MI [13]. Current guidelines specifically advocate for serial ECGs if the initial ECG is normal or non-diagnostic while the patient remains symptomatic and suspicion for MI persists. This allows the identification of evolving ischaemic changes. 

## Conclusions

The likelihood of MI in a young patient presenting to the A&E department with chest pain is relatively low. However, it is crucial to consider the possibility of atypical presentations, as highlighted in this case, particularly given the patient’s young age, atypical pain characteristics, and the absence of significant past medical or family history. This case underscores the value of early troponin testing, serial ECGs and having a high degree of clinical vigilance in accurately identifying MIs. 
